# Artificial intelligence in dentistry—A review

**DOI:** 10.3389/fdmed.2023.1085251

**Published:** 2023-02-20

**Authors:** Hao Ding, Jiamin Wu, Wuyuan Zhao, Jukka P. Matinlinna, Michael F. Burrow, James K. H. Tsoi

**Affiliations:** ^1^ Applied Oral Sciences & Community Dental Care, Faculty of Dentistry, The University of Hong Kong, Pokfulam, Hong Kong SAR, China; ^2^ Division of Dentistry, School of Medical Sciences, The University of Manchester, Manchester, United Kingdom; ^3^ Restorative Dental Sciences, Faculty of Dentistry, The University of Hong Kong, Pokfulam, Hong Kong SAR, China

**Keywords:** artficial intelligence (AI), machine learning, neural network, dentistry, evidence-based dentistry

## Abstract

Artificial Intelligence (AI) is the ability of machines to perform tasks that normally require human intelligence. AI is not a new term, the concept of AI can be dated back to 1950. However, it did not become a practical tool until two decades ago. Owing to the rapid development of three cornerstones of current AI technology—big data (coming through digital devices), computational power, and AI algorithm—in the past two decades, AI applications have started to provide convenience to people's lives. In dentistry, AI has been adopted in all dental disciplines, i.e., operative dentistry, periodontics, orthodontics, oral and maxillofacial surgery, and prosthodontics. The majority of the AI applications in dentistry are for diagnosis based on radiographic or optical images, while other tasks are not as applicable as image-based tasks mainly due to the constraints of data availability, data uniformity, and computational power for handling 3D data. Evidence-based dentistry (EBD) is regarded as the gold standard for decision making by dental professionals, while AI machine learning (ML) models learn from human expertise. ML can be seen as another valuable tool to assist dental professionals in multiple stages of clinical cases. This review describes the history and classification of AI, summarizes AI applications in dentistry, discusses the relationship between EBD and ML, and aims to help dental professionals better understand AI as a tool to support their routine work with improved efficiency.

## 1. Introduction

The fourth industrial revolution is opening a new era, one of the most important contributions of which is Artificial Intelligence (AI). With more and more electronic devices assisting people's life comprehensively, it has become possible to use and analyze the data from these devices through AI. AI is blossoming and expanding rapidly in all sectors. It can learn from human expertise and undertake works typically requiring human intelligence. One of its definitions (1) is “*the theory and development of computer systems able to perform tasks normally requiring human intelligence, such as visual perception, speech recognition, decision making, and translation between languages*”.

AI has been adopted in many fields of industry, such as robots, automobiles, smart city, and financial analysis, *etc*. It has also been used in medicine and dentistry, for example, medical and dental imaging diagnostics, decision support, precision and digital medicine, drug discovery, wearable technology, hospital monitoring, robotic and virtual assistants. In many cases, AI can be regarded as a valuable tool to help dentists and clinicians reduce their workload. Besides diagnosing diseases using a single information source directed at a specified disease, AI can learn from multiple information sources (multi-modal data) to diagnose beyond human capabilities. For example, fundus photographs with other medical data such as age, gender, BMI, smoking habits, blood pressure, and the likelihood of diabetes has been used to predict heart disease (2). Thus, the AI can discover not only eye diseases such as diabetic retinopathy from fundus photography, but also heart disease. It looks like image-based analysis using AI is sound and successful. All these rely on the rapid development (as an output) of computing capacity (hardware), algorithmic research (software), and large database (input data). Given these, there is great potential for the use of AI in the dental and medical field.

Many studies on AI applications in dentistry are underway or even have been put into practise in the aspects such as diagnosis, decision-making, treatment planning, prediction of treatment outcome, and disease prognosis. Many reviews regarding dental AI (3–8) have been published, while this review aims to narrate the development of AI from incipient stages to present, describe the classifications of AI, summarise the current advances of AI research in dentistry, and discuss the relationship between Evidence-based dentistry (EBD) and AI. Limitations of the current AI development in dentistry are also discussed.

## 2. Artificial intelligence

### 2.1. History of AI

Artificial intelligence is not a new term. Alan Turing wrote in his paper “Computing Machinery and Intelligence” (9) in the 1950 issue of *Mind*:

*“I believe that at the end of the century (20th), the use of words and general educated opinion will have altered so much that one will be able to speak of machines thinking without expecting to be contradicted.”*

Back then, there was no term to interpret AI; Turing described AI as “machines thinking”. He mathematically investigated the feasibility of AI and explored how to construct intelligent machines and assess machine intelligence. He proposed that humans solve problems and make decisions by utilising available information and inference, machines also can do the same thing.

In the paper (9), Turing proposed setting a test as to whether a machine can achieve human-level intelligence. This test is known as the Turing Test. It lies on the following lines: Assuming a human evaluator could distinguish natural language communications between a human test taker and a machine. It is given that a human evaluator knows that the conversation is between a human and a machine, and the human evaluator, human test taker and machine are separated from one another. The conversation between the human test taker and the machine is limited to plain text, i.e., keyboard input, instead of speech. This is to make the test only focus on the machine's ability to answer the questions logically instead of testing its speech interpretation ability. If the human evaluator cannot distinguish the human test taker and the machine, the machine can be viewed as having passed the Turing Test, and such a machine is said to have “machine intelligence”.

Later, in 1955, the term AI was first proposed in a 2-month workshop: *Dartmouth Summer Research Project on Artificial Intelligence* (10) led by John McCarthy, Marvin Minsky, Nathaniel Rochester, and Claude Shannon. However, the concept was only on paper. Certain restrictions stopped researchers from developing real AI machines in the 1950s. Firstly, computers before 1949 lacked a fundamental prerequisite for AI tasks: there was no storage function, which means the codes could not be stored, they could only be executed. Secondly, computers were costly at that time. Lastly, funding sources had conservative attitudes towards this new field back then (11).

From 1957 to 1974, the AI field was fast-growing because of the growth of computer power, its accessibility, and AI algorithms. Examples include ELIZA, a computer program that could interpret spoken language and solve problems *via* text (12). Two “AI Winters” arrived after the first wave of development due to insufficient practical applications and research funding reduction in the mid-1970s and late 1980s (13). However, AI had its breakthrough between the two periods with very few developments. In the 1980s, it developed through two paths: machine learning (ML) and expert systems. Theoretically, these are two opposite approaches to AI. ML allows computers to learn by experience (14); expert systems, on the contrary, simulate the decision-making process of human experts (15). In other words, ML finds the solution by learning and summarizing the experience by itself, while expert systems need human experts to input all possible situations and solutions in advance. Expert systems have largely been used in industry since then. The example includes R1 (Xcon) program, an expert system with around 2,500 rules for assisting component selection for computer assembly was developed (16) and used by DEC, a computer manufacturer.

Two important time points in computer vision are 2012 and 2017. In 2012, a graphics processing unit (GPU)-implemented deep learning (DL) network with eight layers was developed (17), The work won the ImageNet Large Scale Visual Recognition Challenge (ILSVRC) and achieved a classification top-5 error of 15.3%. The error rate was more than 10.8% lower than the runner-up. In 2017, SE-NET further lowered the top-5 error to 2.25%, surpassing the human top-5 error (5.1%) (18).

Later famous AI examples include Deep Blue—a chess-playing expert system, which defeated chess champion of the time Gary Kasparov in 1997 (19); 20 years later in 2017, Google's AlphaGo, a DL program, defeated the world No. 1 ranked player Jie Ke in a Go match (20); recently in late 2022, OpenAI launched ChatGPT (Chat Generative Pre-trained Transformer), it is a text-generation model that can generate human-like responses based on text input, the model received extensive discussion since its launch (21). These examples used different AI approaches to operate.

### 2.2. Classification of AI

There are many approaches to achieving AI: different types of AI can achieve different tasks, and researchers have created different AI classification methods.

**Figure 1:**
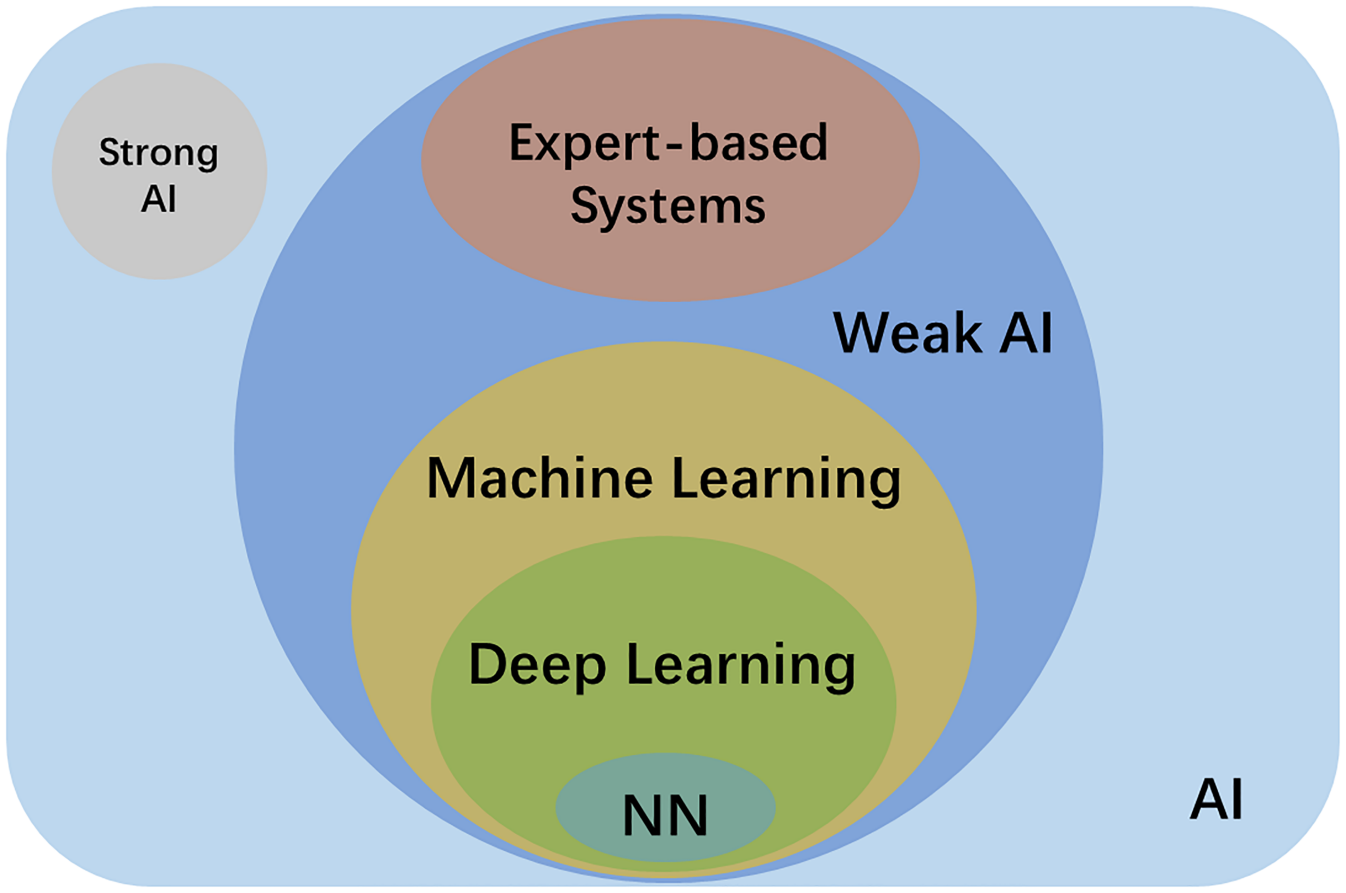
Schematic diagram of the relationship between AI, strong AI, weak AI, expert-based systems, machine learning, deep learning and neural network (NN).

AI is a generic term for all non-human intelligence. As Figure 1 shows, AI can be further classified as weak AI and strong AI. Weak AI, also called narrow AI, uses a program trained to solve single or specific tasks. The AI of today is mostly weak AI. Examples include reinforcement learning, e.g., AlphaGo, and automated manipulation robots; natural language processing, e.g., Google translation, and Amazon chat robots computer vision, e.g., Tesla Autopilot, and face recognition; data mining, e.g., market customer analysis and personalised content recommendation on social media (22). Strong AI refers to the ability and intelligence of AI equalling that of humans—it has its own awareness and behaviour as flexible as humans (23). Strong AI aims to create a multi-task algorithm to make decisions in multiple fields. Research on strong AI has to be very cautious as there might be ethical issues, and it could be dangerous. Thus, there are no strong AI applications up to now.

ML and expert systems are two different subgroups of weak AI. As shown in Table 1, ML can be further classified as supervised, semi-supervised and unsupervised learning based on the theory of the methods. Supervised learning uses labelled datasets for training, and these labelled datasets are the “supervisor” of the algorithm. The algorithm learns from the labelled input, and extracts and identifies the common features of the labelled input to make predictions about unlabelled input (24). Examples of supervised learning include k-nearest neighbors, logistic regression, random forest, and support-vector machines (25). Unsupervised learning, on the contrary, works on its own to find the various features of unlabelled data (26). Semi-supervised learning lies between those two, which utilises a small amount labelled data together with a large amount of unlabelled data during training (27). Recently, a new method called weakly-supervised learning became increasingly popular in the AI field to alleviate labelling costs. In particular, the object segmentation task only uses image-level labels (i.e., only knowing what objects are in the images) instead of object boundary or location information for training (28).

**Table 1:** A comparison of supervised learning, semi-supervised learning, and unsupervised learning.

Items	Supervised learning	Semi-supervised learning	Unsupervised learning
Input type	Labelled data	A mixture of labelled and unlabelled data	Unlabelled data
Accuracy	High	Mid	Low
Complexity of the algorithm	Low	Mid	High
Types of algorithm	Regression and classification	Regression, classification, clustering, and association	Clustering and association

**Figure 2:**
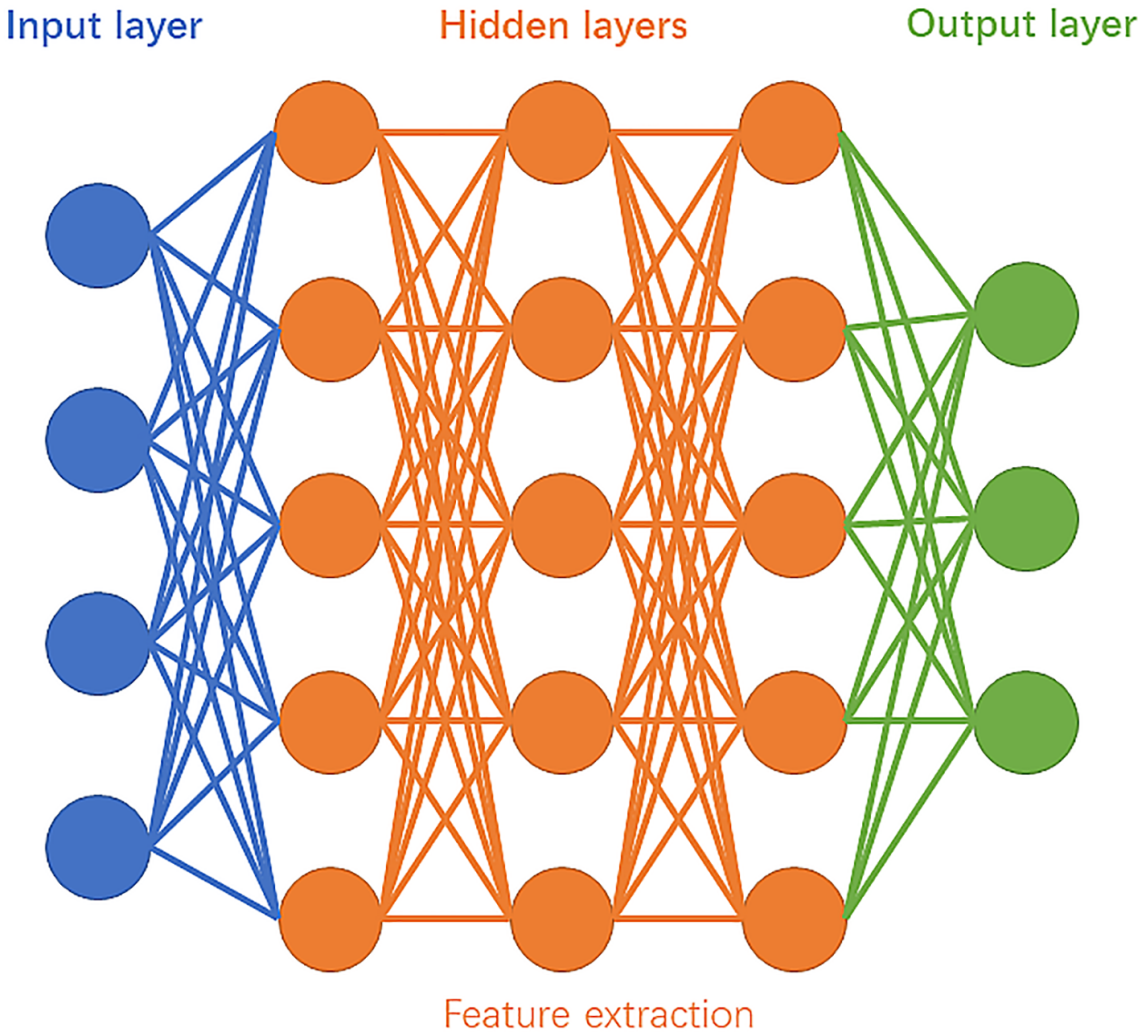
Schematic diagram of deep learning.

Deep learning is currently a very prominent research area and forms a subset of ML. It can involve both supervised and unsupervised learning. As Figure 2 shows, “deep” represents an artificial “neural network” consisting of a minimum of three nodal layers—input, multiple “hidden”, and output layers such that each layer consists of various numbers of interconnected nodes (artificial neurons) whereas each node *x* has an associated weight (*w_i_*) and biased threshold (*t*) from *m* decisive factors, given by its own (simplified) linear regression model . The weight is assigned when there is an input of the node. If $\sum_{i=1}^{m}w_{i}x_{i}+t\geq 0$, then the output = 1, meaning the data is passed to another node in another layer. The process of passing data from one layer to the next defines the neural network as a feedforward network, similar to a decision tree model.

As mentioned above, a deep neural network can extract features from the imported data, which does not require human intervention. Instead, it can learn those features from large datasets. On the other hand, expert systems require human intervention to learn, which indeed tuning the *w_i_* and *t* manually. So, less data is required.

Neural networks (NNs) are biologically inspired networks that can be regarded as the pillars of deep learning algorithms. There are different variations of NNs, among which the most important types of neural networks are artificial neural networks (ANNs), neural networks (CNNs), and generative adversarial networks (GANs).

#### 2.2.1. ANN

ANN comprises a group of neurons and layers, as illustrated in Figure 2. As mentioned above, this model is a basic model for deep learning, consisting of a minimum of three layers. The inputs are processed only in the forward direction. Input neurons extract features of input data from the input layer and send data to hidden layers, and the data goes through all the hidden layers successively. Finally, the results are summarised and shown in the output layer. All the hidden layers in ANN can weigh the data received from previous layers and make adjustments before sending the data to the next layer. Each hidden layer acts as an input and output layer, allowing the ANN to understand more complex features (29).

#### 2.2.2. CNN

CNN is a type of deep learning model mainly used for image recognition and generation. The mean difference between ANN and CNN is that CNN consists of convolution layers, in addition to the pooling layer and the fully connected layer in the hidden layers. Convolution layers are used to generate feature maps of input data using convolution kernels. The input image is folded by the kernels completely. It reduces the complexity of images because of the weight sharing by convolution. The pooling layer is usually followed by each group of convolution layers, which reduces the dimension of feature maps for further feature extraction. The fully connected layer is used after the convolution layer and pooling layer. As the name indicates, the fully connected layer connects to all activated neurons in the previous layer and transforms the 2D feature maps into 1D. 1D feature maps are then associated with nodes of categories for classification (30, 31). By using the above-mentioned functional hidden layers, CNN showed higher efficiency and accuracy in image recognition compared with ANN.

#### 2.2.3. GAN

GAN is one kind of deep learning algorithm designed by Goodfellow et al*.* (32) in 2014. It is an unsupervised learning method designed to automatically discover patterns from the input data and generate new data with similar features or patterns compared with the input data. GAN consists of two neural networks: a generator and a discriminator. The ultimate goal for the generator is to generate data such that the discriminator cannot determine whether the data is generated by the generator or from the original input data. The ultimate goal for the discriminator is to distinguish the generator-generated data from the original input data as much as possible. The two networks compete with each other in GAN, and both networks improve themselves during the competition.

Since GAN was designed, the network has rapidly spread in AI applications. They are mainly applied to image-to-image translation and generating plausible photos of objects, scenes, and people (33, 34). Wu et al*.* (35) proposed a new 3D-GAN framework in 2016 based on a traditional GAN network. 3D-GAN generates 3D objects from a given 3D space by combining recent advances in GAN and volumetric convolutional networks. Unlike a traditional GAN network, it can generate objects in 3D directly or from 2D images. It gives a broader range of possible applications in 3D data processing compared with its 2D form.

## 3. AI in dentistry

As in other industries, AI in dentistry has started to blossom in recent years. From a dental perspective, applications of AI can be classified into diagnosis, decision-making, treatment planning, and prediction of treatment outcomes. Among all the AI applications in dentistry, the most popular one is diagnosis. AI can make more accurate and efficient diagnoses, thus reducing dentists' workload. On one hand, dentists are increasingly relying on computer programs for making decisions (36, 37). On the other hand, computer programs for dental use are becoming more and more intelligent, accurate, and reliable. Research on AI has spread over all fields in dentistry.

Although a large amount of journal articles regarding dental AI have been published, it is still difficult to compare between articles in terms of study design, data allocation (i.e., training, test, and validation sets), and model performance (i.e., accuracy, sensitivity, specificity, F1, AUC {Area Under [the receiver operating characteristic (ROC)] Curve}, recall). Most articles failed to report the information mentioned above entirely. Thus, the MI-CLAIM (Minimum Information about Clinical Artificial Intelligence Modeling) checklist has been advocated to bring similar levels of transparency and utility to the application of AI in medicine (38).

### 3.1. AI in operative dentistry

Traditionally, dentists diagnose caries by visual and tactile examination or by radiographic examination according to a detailed criterion. However, detecting early-stage lesions is challenging when deep fissures, tight interproximal contacts, and secondary lesions are present. Eventually, many lesions are detected only in the advanced stages of dental caries, leading to a more complicated treatment, i.e., dental crown, root canal therapy, or even implant. Although dental radiography (whether panoramic, periapical, or bitewing views) and explorer (or dental probe) have been widely used and regarded as highly reliable diagnostic tools detecting dental caries, much of the screening and final diagnosis tends to rely on dentists' experience.

In operative dentistry, there has been research on the detection of dental caries, vertical root fractures, apical lesions, pulp space volumetric assessment, and evaluation of tooth wear (39–44) (Table 2). In a two-dimensional (2D) radiograph, each pixel of the grayscale image has an intensity, i.e., brightness, which represents the density of the object. By learning from the above-mentioned characteristics, an AI algorithm can learn the pattern and give predictions to segment the tooth, detect caries, *etc*. For example, Lee et al*.* (45) developed a CNN algorithm to detect dental caries on periapical radiographs. Kühnisch et al*.* (46) proposed a CNN algorithm to detect caries on intraoral images. Schwendicke et al*.* (47) compared the cost-effectiveness of AI for proximal caries detection with dentists' diagnosis; the results showed that AI was more effective and less costly.

**Table 2:** Examples of AI applications in operative dentistry.

Study	Type of data	Type of algorithm	Size of dataset (training/testing)	Accuracy	Sensitivity	Specificity	AUC	Other performances
Vertical root fracture detection (40)	Panoramic radiography	CNN	240/60		0.75			Precision: 0.93; F1: 0.83
Apical lesion detection (42)	CBCT images	CNN	16/4		0.93	0.88		PPV: 0.87; NPV: 0.93
Tooth wear evaluation (43)	Patient's information and oral conditions, intraoral optical images	SVM, KNN	245 in total	SVM: 0.69 KNN: 0.48				
Dental caries detection (45)	Periapical radiography	CNN	2400/600	0.82–0.89	0.81–0.923	0.83–0.94	0.845–0.917	
Dental caries detection (46)	Intraoral optical images	CNN	1891/479	92.5%–93.3%	0.896–0.957	0.815–0.943	0.955–0.964	

AUC, Area under [the receiver operating characteristic (ROC)] Curve; CBCT, cone-beam computed tomography; CNN, convolutional neural network; KNN, K-Nearest neighbor; NPV, negative predictive value; PPV, positive predictive value; SVM, support-vector machine.

Several studies mentioned above showed that AI has promising results in early lesion detection, with the same accuracy or even better compared with dentists. This achievement requires interdisciplinary cooperation between computer scientists and clinicians. The clinicians manually label the radiographic images with the location of caries while the computer scientists prepare the dataset and ML algorithm. Finally, clinicians and computer scientists jointly check and verify the accuracy and precision of the training results (48).

### 3.2. AI in periodontics

Periodontitis is one of the most widespread diseases. It is a burden for billions of individuals and, if untreated, can lead to tooth mobility and even tooth loss (49). To prevent severe periodontitis, early detection and treatment are needed. In clinical practise, periodontal disease diagnosis is based on evaluating pocket probing depths and gingival recession. The Periodontal Screening Index (PSI) is frequently used to quantify clinical attachment loss. However, this clinical evaluation has low reliability: the screening for periodontal disease is still based on the experience of dentists, and they may miss localized periodontal tissue loss (50).

In periodontics, AI has been utilised to diagnose periodontitis and classify plausible periodontal disease types (51, 52). In addition, Krois et al*.* (50) adopted CNN in the detection of periodontal bone loss (PBL) on panoramic radiographs. Lee et al*.* (53) evaluated the potential usefulness and accuracy of a proposed CNN algorithm to detect periodontally compromised teeth automatically. Yauney et al*.* (54) claimed that periodontal conditions could be examined by a CNN algorithm developed by their research group using systemic health-related data (Table 3).

**Table 3:** Examples of AI applications in periodontics.

Study	Type of data	Type of algorithm	Size of dataset (training/testing)	Accuracy	Sensitivity	Specificity	AUC	Other performances
Periodontal bone loss detection (50)	Panoramic radiography	CNN	1456/353	0.81	0.81	0.81	0.89	F1: 0.78; PPV: 0.76; NPV: 0.85
Severity of chronic periodontitis prediction (51)	Bacterial category in subgingival biofilms, Patient's information and oral conditions	NN, RF, SVM, RLR	692/45	NN: 0.80–0.93 RF: 0.78–0.93 SVM: 0.78–0.92 RLR: 0.79–0.92	NN: 0.67–0.95; RF: 0.71–0.96; SVM: 0.72–0.97; RLR: 0.75–0.97	NN: 0.79–0.88; RF: 0.72–0.83; SVM: 0.61–0.82; RLR: 0.64–0.81	NN: 0.82–0.96; RF: 0.81–0.96; SVM: 0.83–0.96; RLR: 0.82–0.97	
Periodontally compromised teeth detection (53)	Periapical radiography	CNN	1392/348	0.734–0.828			0.734–0.826	
Periodontal condition examination (54)	Systemic health-related data, intraoral optical images	CNN	284 in total		0.429		0.677	Precision: 0.271

AUC, Area under the ROC curve; CNN, convolutional neural network; NN, neural network; NPV, negative predictive value; PPV, positive predictive value; RF, random forest; RLR, regularized logistic regression; SVM, support-vector machine.

### 3.3. AI in orthodontics

Orthodontic treatment planning is usually based on the experience and preference of the orthodontists. As every patient and orthodontist is unique, the treatment is decided mutually by both sides. Traditionally, it takes a lot of effort for orthodontists to diagnose malocclusion, as many variables need to be considered in the cephalometric analysis, such that it is difficult to determine the treatment plan and predict the treatment outcome (55). AI is an ideal tool for solving orthodontic problems. In orthodontics, AI has applications (Table 4) in treatment planning and prediction of treatment results, such as simulating the changes in the appearance of pre- and post-treatment facial photographs. The impact of orthodontic treatment, the skeletal patterns, and the anatomic landmarks in lateral cephalograms (67) can be clearly seen with the aid of AI algorithms, greatly assisting communication between patients and dentists.

**Table 4:** Examples of AI applications in orthodontics.

Study	Type of data	Type of algorithm	Size of dataset (training/testing)	Accuracy	Sensitivity	Specificity	AUC	Other performances
Orthodontic treatment results prediction (56)	Facial 3D images	DL	137 in total	N/A				
Diagnosis of the need for orthodontic treatment (57)	Orthodontics-related oral condition data	Bayesian network	800/200	0.93–0.96	0.94–0.96	0.94–1	0.91	
Tooth extraction determination in orthodontic treatments (58)	Orthodontics-related indices	ANN	180/20	0.8				
Tooth extraction determination in orthodontic treatments (59)	Cephalometric variables, orthodontics-related indices	ANN	96/60	0.93				ICC: 0.97–0.99
Cephalometric landmarks locating (60, 61)	Lateral cephalometric radiography	CNN	1028/283	0.804–0.962				
Tooth landmark/axis detection (62)	Intraoral optical images, CBCT images	NN	2219/865					Average errors: 0.37 mm (landmark detection); 3.33° (axis detection)
Skeletal classification (63)	Lateral cephalometric radiography	CNN	5890 in total	0.8951–0.964	0.8427–0.9459	0.9213–0.9729	0.889–0.991	
Tooth surgery/extraction determination in orthodontic treatments (64)	Lateral cephalometric radiography, orthodontics-related indices	ANN	204/112	0.91–0.96				ICC: 0.97–0.99
Tooth segmentation (65)	3D models from intraoral scanner	CNN	1600/400	0.980–0.986				F1: 0.942
Tooth and alveolar bone segmentation (66)	CBCT images	CNN	3172/1359	Tooth: 0.915 Alveolar bone: 0.93	Tooth: 0.921; Alveolar bone: 0.935			

3D, three*-*dimensional; AUC, Area under the ROC curve; ANN, artificial neural network; CBCT, cone-beam computed tomography; CNN, convolutional neural network; DL, deep learning; NN, neural network; ICC, intraclass correlation coefficient.

A Bayesian-based decision support system was developed by Thanathornwong (57) to diagnose the need for orthodontic treatment based on orthodontics-related data as input. Xie et al*.* (58) proposed an ANN model to evaluate whether extractions are needed from lateral cephalometric radiographs; A similar evaluation system was proposed by Jung et al*.* (59). Apart from the application in predicting the extractions needed for orthodontic purposes, AI has been adopted to locate cephalometric landmarks. Park et al*.* (60, 61) demonstrated a DL algorithm for the automatically identifying cephalometric landmarks on radiographs with a high accuracy. Bulatova (68) et al*.* and Kunz et al*.* (69) developed similar AI algorithms, with accuracies comparable with human examiners in identifying those landmarks. An automatic system for skeletal classification using lateral cephalometric radiographs was proposed by Yu et al*.* (63).

Besides locating multiple cephalometric landmarks and classification, AI systems have been used in orthodontic treatment planning. Choi et al*.* (64) proposed an AI model to judge whether surgery is needed using lateral cephalometric radiographs. It looks like most of the orthodontic applications are on landmarking identification and treatment planning, which are tedious procedures for orthodontists. A basic task for orthodontic treatment planning is to segment and classify the teeth. AI has also been used for these purposes on multiple sources, such as radiographs and full-arch 3D digital optical scans (65, 66). Cui et al*.* proposed several AI algorithms to automatically segment teeth on a digital teeth model scanned by a 3D intraoral scanner (65) and CBCT images (66, 70). In addition to tooth segmentation, they also segmented alveolar bone, the efficiency exceeded the radiologists' work (i.e., 500 times faster). The paper also claimed that the algorithm works well in challenging cases with variable dental abnormalities (66).

### 3.4. AI in oral and maxillofacial pathology

Oral and Maxillofacial Pathology (OMFP) is a specialty for examining pathological conditions and diagnosing diseases in the oral and maxillofacial region. The most severe type of OMFP is oral cancer. Statistics from the World Health Organization (WHO) show that every year there are over 657,000 patients diagnosed with oral cancer globally, among which there are more than 330,000 deaths (71). In OMFP, as shown in Table 5, AI has been researched mostly for tumour and cancer detection based on radiographic, microscopic and ultrasonographic images. In addition, AI can be used to detect abnormal sites on radiographs (72), such as nerves in the oral cavity, interdigitated tongue muscles, and parotid and salivary glands. CNN algorithms were demonstrated to be a suitable tool for the automatically detecting cancers (73, 78). It is worth mentioning that AI also plays a role in managing cleft lip and palate in risk prediction, diagnosis, pre-surgical orthopaedics, speech assessment, and surgery (79).

**Table 5:** Examples of AI applications in oral and maxillofacial surgery.

Study	Type of data	Type of algorithm	Size of dataset (training/testing)	Accuracy	Sensitivity	Specificity	AUC	Other performances
Mandibular third molar and IAN positional relationship detection (72)	Panoramic radiography	CNN (ResNet-50)	571 in total	0.7232–0.8065	0.8462–0.8667	0.5532–0.75	0.66–0.83	Precision: 0.62–0.83; F1: 0.61–0.73
OSCC diagnosis (73)	Confocal laser endomicroscopy images	CNN	116 video sequences	0.883	0.866	0.9	0.96	
OPMDs and OSCC diagnosis (74)	Intraoral optical images	CNN	980 in total		0.73–0.99	0.83–0.99	0.71–1	Precision: 0.63–0.98; F1: 0.68–0.98
OPMDs diagnosis (75)	OCT Images	ANN, SVM	128/271 sets	0.52–0.84	0.83–0.93	0.69–0.82		PPV: 0.51–0.95; NPV: 0.76–0.96
OPMDs diagnosis (76)	OCT Images	CNN	6/15 sets	0.82	1	0.7		
Ameloblastoma and KCOT diagnosis (77)	Panoramic radiography	CNN	400/100	0.83	0.818	0.833	0.88	Diagnostic time: 38 s

AUC, Area under the ROC curve; CNN, convolutional neural network; IAN, inferior alveolar nerve; KCOT, keratocystic odontogenic tumour; NPV, negative *p*redictive value; OCT, optical coherence tomography; OPMD, oral potentially malignant disorder; OSCC: oral squamous cell carcinoma; PPV, positive predictive value.

Early detection and diagnosis of various mucosal lesions are essential to classify them as benign or malignant. Surgery resection is required for malignant lesions. However, some of the lesions behave similarly in appearance, thus requiring the diagnosis by biopsy slides and radiographs. Pathologists diagnose disease by observing the morphology of stained specimens on glass slides using a microscope (80). It is tedious work that requires much of effort for pathologists. Of all the biopsies that need to be examined, only around 20% of them are found to be malignancies. Thus, AI can be a suitable tool for aiding pathologists in this task.

Warin et al*.* (74) used a CNN approach to detect oral potentially malignant disorders (OPMDs) and oral squamous cell carcinoma (OSCC) in intraoral optical images. In addition to intraoral optical images, OCT has been used in identify benign and malignant lesions in the oral mucosa. James et al*.* (75) used ANN and SVM models to distinguish malignant and dysplastic oral lesions. Heidari et al*.* (76) used a CNN network, AlexNet (17), to distinguish normal and abnormal head and neck mucosa. Abureville et al*.* (73) used a CNN algorithm to automatically diagnose oral squamous cell carcinoma (SCC) from confocal laser endomicroscopy images; the study showed that the CNN algorithm used in the study was especially suitable for early diagnosis of SCC. Poedjiastoeti et al*.* (77) also used a CNN algorithm to identify and distinguish ameloblastoma and keratocystic odontogenic tumour (KCOT). The two oral tumours with similar features in radiographic images. By comparing the computer-generated results with the biopsy results, the accuracy of the CNN algorithm was found to be 83% and the diagnostic time 38 s. These values were similar to those of oral and maxillofacial specialists.

### 3.5. AI in prosthodontics

In prosthodontics, a typical treatment process to prepare a dental crown includes tooth preparation, impression taking, cast trimming, restoration design, fabrication, try-in, and cementation. The application of AI in prosthodontics mainly lies in the restoration design (Table 6). CAD/CAM has digitalised the design work in commercialized products, including CEREC, Sirona, 3Shape, *etc*. Although this has dramatically increased the efficiency of the design process by utilising a tooth library for crown design, it still cannot achieve a custom-made design for individual patients (81). With the development of AI, Hwang et al*.* (82) and Tian et al*.* (83) proposed novel approaches based on 2D-GAN models to generate a crown by learning from technicians' designs. The training data was 2D depth maps converted from 3D tooth models. Ding (84) reported a 3D-DCGAN network in the crown generation, which utilised 3D data directly in the crown generation process, the morphology of generated crowns was similar compared with natural teeth. Integrating AI with CAD/CAM or 3D/4D printing can achieve a more desirable workflow with high efficiency (88). AI has also been used in shade matching (85) and debonding prediction of CAD/CAM restorations (86).

**Table 6:** Examples of AI applications in prosthodontics.

Study	Type of data	Type of algorithm	Size of dataset (training/testing)	Accuracy	Sensitivity	Specificity	AUC	Other performances
Crown generation (82)	Intraoral scanner/depth map	GAN	3070/243 (Teeth)					
Crown generation (83)	Intraoral scanner/depth map	GAN	700/80 (Teeth)					
Crown generation (84)	Intraoral scanner	3D–DCGAN	600/12					
Shade matching (85)	CIE LAB color space number	BPNN	39/4					The proposed method had a lower *Δ*E compared with traditional visual shade matching.
Resin composite crowns debonding prediction (86)	optical images of abutments	CNN	6480/2160	0.985	1		0.998	Precision: 0.97; F1: 0.985
Dental arch classification (87)	Intraoral optical images	CNN	1016/168	0.995–0.997	1		0.98–0.99	Precision: 0.25 F1: 0.4

AUC, Area under the ROC curve; BPNN, back-propagation neural network; CIE, international commission on illumination; CNN, convolutional neural network; GAN, generative adversarial network; 3D-DCGAN, 3-dimensional deep convolutional generative adversarial network.

Apart from fixed prosthodontics, the design in removable prosthodontics is more challenging as more factors and variables need to be considered. No ML algorithm is available for the purpose of designing removable dentures while several expert (knowledge based) systems have been introduced (89–91). Current ML algorithms are more focused on assisting the design process of removable dentures, e.g., classification of dental arches (87), and facial appearance prediction in edentulous patients (92).

## 4. Discussion

Given the success of AI, it has been proved that AI can learn beyond human expertise. In fact, the development of AI cannot be achieved without the development of computer technology (software), computing capacity (hardware), and large database (input data). ML tasks involving 3D models require high computational power to train the algorithm. Current computational power may be still insufficient to work directly on 3D data to perform classification or regression tasks compared with well-studied 2D image and video-based tasks. Millions of point clouds or meshes in a 3D model cannot be loaded to GPU at once. Sampling and representations of a 3D model (i.e., depth map, voxels, point cloud, and mesh) are often used to reduce the computation burden, such that the details would be sacrificed during the transition. In addition to the massive amount of digitalised medical data used for training ML models, which did not exist previously, the development of wearable devices also contributes to the acquisition of medical big data. Thus, the evolution of AI applications is greatly dependent on the AI algorithm, computational power, and digitalised training data.

Evidence-Based Dentistry (EBD), a more specific branch of Evidence-Based Medicine (EBM), is defined as “*an approach to oral health care that requires the judicious integration of systematic assessments of clinically relevant scientific evidence, relating to the patient's oral and medical condition and history, with the dentist's clinical expertise and the patient's treatment needs and preferences*” (93). Both EBM and EBD are regarded as the gold standard for the decision-making of health professionals. While ML models learn from human expertise, this can be seen as another useful tool for health professionals in multiple stages of clinical cases.

On one hand, ML could assist clinicians in storing and analysing constantly updated medical knowledge and patient-related data. ML algorithms are adept at finding patterns in patients' diagnostic data, improving current medical treatment, discovering new drugs, precision medicine, and minimising human error. EBD has a similar aim, but ML can finish it more quickly as it uses existing data, while EBD usually needs randomized controlled trials to achieve those aims. On the other hand, medical data are challenging to handle since the diagnosis is usually based on multiple sources. ML requires a large amount of data for training which may be subject to systematic bias or be inaccessible; these could influence the ultimate result. It is not easy to improve the precision of a ML model by only increasing the training data instead of increasing the quality of the data. Also, ML cannot account for the differing diagnoses by different clinicians using different data sources.

In addition, medical data are often stored within isolated, individualised, and limitedly interoperable systems due to concerns such as ethical problems, data protection, and organisational barriers. The research on federated learning (94) of ML is a potential way to solve data privacy protection problems. Besides, professional personnel are usually required to label dental and medical data. These limitations lead to the datasets lacking structure and insufficient, at least when compared with other AI fields (95). Few-shot learning has been studied to tackle this problem (96).

To use dental and medical data for ML training, one must be very careful with its complex, sensitive, and limited validation methods (97). Dental and medical data from electronic records are usually of low integrity. The data often lack of systematic allocation and is not at random, e.g., data from the hospital may have a risk of being overly sick; data collected from wearable devices may have a risk of being overly healthy. Furthermore, healthcare system level in different countries or regions is unbalanced. Data from one single country or region could possibly lead to the training result being precise but not accurate and cannot apply to countries with different healthcare system conditions. AI applications trained by such data will be biased (95). ML using such long-tailed data have been studied to minimise its influence (98). Besides, the outcomes of AI are often not readily applicable. The single output provided by most contemporary medical AI applications will only partially inform the required and complex decision-making of clinical applications. Unlike EBD, ML does not have a system to monitor the quality of the input medical data and the degree of bias. EBD has a more macroscopic awareness, and decisions are usually made based on several data sources to minimise bias. Due to the above-mentioned constraints, some clinicians have reserved their opinion on ML due to its “black box” mechanism, which the rationale for getting to the specific results cannot be explained. Although explainable AI has been studied for this purpose (99), EBD is straightforward and has a more transparent mechanism (100).

EBD and ML have their own advantages and disadvantages. ML is a new approach in the medical field to improve diagnosis and predict treatment outcomes by discovering patterns and associations amongst medical datasets. However, while current ML applications mainly rely on the same type of dataset, ML is capable of acquiring information from EBD, which uses different kinds of data for diagnosis. EBD can also benefit from the addition of ML in facilitating the discovery of the underlying connection between medical data and disease and in providing a better and individualised diagnosis. EBD and ML are complementary to serve clinicians better; clinicians can refer to both to maximise their advantage and apply them to medical practise.

## 5. Conclusion

New technologies are developed and adopted rapidly in the dental field. AI is among the most promising ones, with features such as high accuracy and efficiency if unbiased training data is used and an algorithm is properly trained. Dental practitioners can identify AI as a supplemental tool to reduce their workload and improve precision and accuracy in diagnosis, decision-making, treatment planning, prediction of treatment outcomes, and disease prognosis.
